# Effects of Resistance-Associated NS5A Mutations in Hepatitis C Virus on Viral Production and Susceptibility to Antiviral Reagents

**DOI:** 10.1038/srep34652

**Published:** 2016-10-05

**Authors:** Sayuri Nitta, Yasuhiro Asahina, Mami Matsuda, Norie Yamada, Ryuichi Sugiyama, Takahiro Masaki, Ryosuke Suzuki, Nobuyuki Kato, Mamoru Watanabe, Takaji Wakita, Takanobu Kato

**Affiliations:** 1 Department of Virology II, National Institute of Infectious Diseases, Tokyo, Japan; 2 Department of Gastroenterology and Hepatology, Tokyo Medical and Dental University, Tokyo, Japan; 3 Faculty of Medicine, Tokyo Medical and Dental University, Tokyo, Japan; 4 Department of Liver Disease Control, Tokyo Medical and Dental University, Tokyo, Japan; 5 Department of Tumor Virology, Okayama University Graduate School of Medicine, Dentistry, and Pharmaceutical Sciences, Okayama, Japan

## Abstract

Direct-acting antivirals (DAAs) for hepatitis C virus (HCV) have potent anti-HCV effects but may provoke resistance-associated variants (RAVs). In this study, we assessed the characteristics of these RAVs and explored efficacious anti-HCV reagents using recombinant HCV with NS5A from a genotype 1b strain. We replaced the NS5A of JFH1 with that of Con1 (JFH1/5ACon1) and introduced known NS5A inhibitor resistance mutations (L31M, L31V, L31I and Y93H) individually or in combination. Susceptibilities against anti-HCV reagents were also investigated. RAVs with Y93H exhibited high extracellular core antigen levels and infectivity titers. Variants with any single mutation showed mild to moderate resistance against NS5A inhibitors, whereas variants with double mutations at both L31 and Y93 showed severe resistance. The variants with mutations exhibited similar levels of susceptibility to interferon (IFN)-α, IFN-λ1, IFN-λ3 and Ribavirin. Variants with the Y93H mutation were more sensitive to protease inhibitors compared with JFH1/5ACon1. In conclusion, the *in vitro* analysis indicated that the Y93H mutation enhanced infectious virus production, suggesting advantages in the propagation of RAVs with this mutation. However, these RAVs were susceptible to protease inhibitors. Thus, a therapeutic regimen that includes these reagents is a promising means to eradicate these RAVs.

Hepatitis C virus (HCV) infection is a major cause of chronic hepatitis, cirrhosis and hepatocellular carcinoma and results in hepatic disease-associated deaths worldwide^1^. For many years, interferon (IFN) has been the main therapeutic agents for HCV infection. However, the efficacy of IFN-based therapy even with Ribavirin (RBV) is restrictive and provides a sustained virological response rate of only approximately 50%, especially for patients infected with genotype 1 strains^1^,^2^. Recent research advances have resulted in the development of many novel anti-viral reagents, including direct-acting antivirals (DAAs)^2^,^3^. DAAs directly target HCV viral proteins and have strong antiviral effects that lead to a high sustained virological response rate. Several approved DAAs (protease inhibitors, non-structural protein 5A (NS5A) inhibitors, and polymerase inhibitors) are currently available for clinical use. Many clinical studies have shown that these DAA therapies with or without IFN-α dramatically improve the efficacy and achieve a high sustained virological response rate^2^. Among these DAAs, NS5A inhibitors have high potency, are well tolerated, and play a pivotal role in DAA therapies^4^. Despite their potent effects, the major issue with the use of these DAAs is the emergence of resistance-associated variants (RAVs)^5^,^6^,^7^. The amino acid mutations L31M, L31V, L31I and Y93H in NS5A of genotype 1b strains have been reported to confer various levels of resistance to Daclatasvir (DCV) or other NS5A inhibitors^8^,^9^,^10^,^11^. Of these mutations, Y93H is associated with high-level resistance, and variants with this polymorphism have been detected in treatment naïve patients^12^,^13^,^14^,^15^. In clinical studies, lower sustained virological response rates were observed in patients with RAVs to NS5A inhibitors compared with patients without these mutations even under combination therapy with protease and NS5A inhibitors^13^,^16^. Furthermore, these polymorphisms have been reported to remain for a long duration (at least 1 year) after the cessation of DCV treatment^9^,^17^,^18^. Therefore, the characteristics and behavior of HCV variants with these resistance-associated mutations and effective antiviral reagents for these variants need to be identified to establish the best therapeutic strategy.

There are several basic studies for resistant-associated mutations to DAAs including NS5A inhibitors. Most of these studies used subgenomic replicons for the analysis, which have critical limitation to evaluate the HCV life cycle because of lacking infectious virus production^19^. *In vitro* cell culture system for HCV is indispensable to assess the whole life cycle of this virus and the cell culture system of several genotype strains have been developed. However, the efficient cell culture system of genotype 1b strains has not yet been developed. The HCV genotype 2a strain designated JFH1 is the most used strain that can replicate efficiently and produce infectious particles in cell culture^20^. We previously established the cell culture system with JFH1-based recombinant virus by replacement of NS5A with that from genotype 1b strain, Con1 (JFH1/5ACon1)^21^. This HCV cell culture system enabled to evaluate the effects of NS5A of genotype 1b on the HCV life cycle and the susceptibility to the NS5A inhibitor.

In this study, we used a cell culture system with a JFH1-based recombinant virus generated by the replacement with the NS5A from the genotype 1b strain Con1 containing resistance-associated NS5A mutations to assess their effects on the HCV life cycle and the susceptibilities of the viruses to various anti-HCV reagents^21^. We found that the Y93H mutation conferred enhanced infectious virus production but was related to the higher susceptibility to protease inhibitors, although the susceptibilities to other antiviral reagents (IFN-α, -λ1, -λ3, and RBV) were not changed.

## Results

### Characteristics of recombinant HCV and its derivatives with resistance-associated NS5A mutations

**Figure 1:**
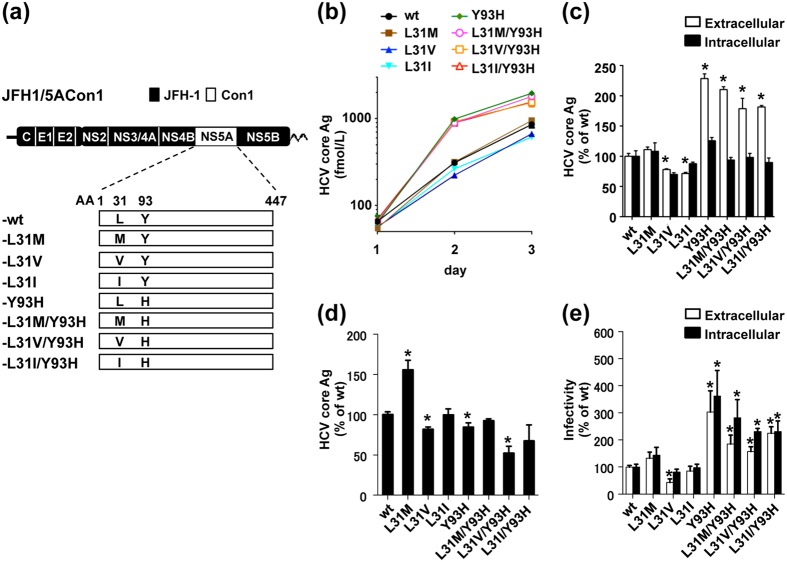
Characteristics of the recombinant virus JFH1/5ACon1 and its derivatives with resistance-associated NS5A mutations. (**a**) Schematic representation of JFH1/5ACon1 and its derivatives with resistance-associated NS5A mutations. (**b**) Amounts of HCV core Ag in the HCV RNA-transfected Huh7.5.1 cells on days 1, 2, and 3 post-electroporation. (**c**) Amounts of HCV core Ag in the culture media and cell lysates were compared on day 3 post-electroporation. (**d**) Amounts of HCV core Ag in the HCV RNA-transfected Huh7-25 cells on day3 post-electroporation. (**e**) Extra- and intracellular infectivity titers of HCV RNA-transfected Huh7-25 cells were measured. (**c**–**e**) The data are presented as percentages of the JFH1/5ACon1-wt data. Statistical significances to JFH1/5ACon1-wt are indicated (*p < 0.05).

To investigate the effect of resistance-associated NS5A mutations on the virus life cycle, we introduced mutations reported in resistance to NS5A inhibitors (L31M, L31V, L31I and Y93H) into the JFH1 based recombinant virus with the NS5A from Con1 (JFH1/5ACon1-wt) individually or in combination to generate JFH1/5ACon1-L31M, -L31V, -L31I, -Y93H, -L31M/Y93H, -L31V/Y93H, and -L31I/Y93H, respectively (Fig. 1a). *In vitro* transcribed full-length HCV RNA from these recombinant viruses was transfected into Huh-7.5.1 cells, and the extracellular HCV core antigen (Ag) levels were measured on days 1, 2, and 3 post-transfection. The HCV core Ag levels in the culture media of these recombinant virus RNA-transfected cells gradually increased in a time-dependent manner, indicating that these recombinant viruses were capable of replicating in Huh-7.5.1 cells (Fig. 1b). At 3 days post-transfection, the intracellular HCV core Ag levels of the variants with mutations were comparable to those of JFH1/5ACon1-wt (Fig. 1c). However, the extracellular HCV core Ag levels of JFH1/5ACon1-L31V and -L31I were slightly lower and the levels in the variants with the Y93H mutation (JFH1/5ACon1-Y93H, -L31M/Y93H, -L31V/Y93H, and -L31I/Y93H) were approximately 2-fold higher than JFH1/5ACon1-wt.

### Effects of resistance-associated NS5A mutations on the HCV life cycle

To determine the steps of the HCV life cycle affected by these resistance-associated NS5A mutations, we exploited the single-cycle virus production assay using Huh7-25 cells^22^,^23^,^24^. This cell line lacks cell surface expression of CD81, which enables the assessment of HCV replication and infectious virus production by removing the influence of re-infection by progeny viruses. *In vitro* transcribed HCV RNA from JFH1/5ACon1 and its derivatives with resistance-associated NS5A mutations was transfected into Huh7-25 cells, and intracellular HCV core Ag levels were measured at 3 days post-transfection to assess the effect on the replication of these variants. As shown in Fig. 1d, the intracellular HCV core Ag level of JFH1/5ACon1-L31M was approximately 1.5-fold higher and the levels of -L31V, -Y93H and -L31V/Y93H were lower than that of JFH1/5ACon1-wt. To assess the effect of these mutations on infectious virus production, we determined the extra- and intracellular infectivity titers of the HCV RNA-transfected Huh7-25 cells. The extra- and intracellular infectivity titer of JFH1/5ACon1-L31M showed no significant difference compared to JFH1/5ACon1-wt (Fig. 1e). The extracellular infectivity titer of JFH1/5ACon1-L31V was lower than that of -wt. In contrast, the extra- and intracellular infectivity titers of variants with Y93H (JFH1/5ACon1-Y93H, -L31M/Y93H, -L31V/Y93H, and -L31I/Y93H) were 1.5- to 3.6-fold higher than those of JFH1/5ACon1-wt. Taken together, these findings suggested that the L31M mutation conferred the virus with enhanced replication, whereas the Y93H mutation promoted the production of infectious virus.

### Susceptibility of JFH1/5ACon1 and its derivatives with resistance-associated NS5A mutations to NS5A inhibitors

**Figure 2:**
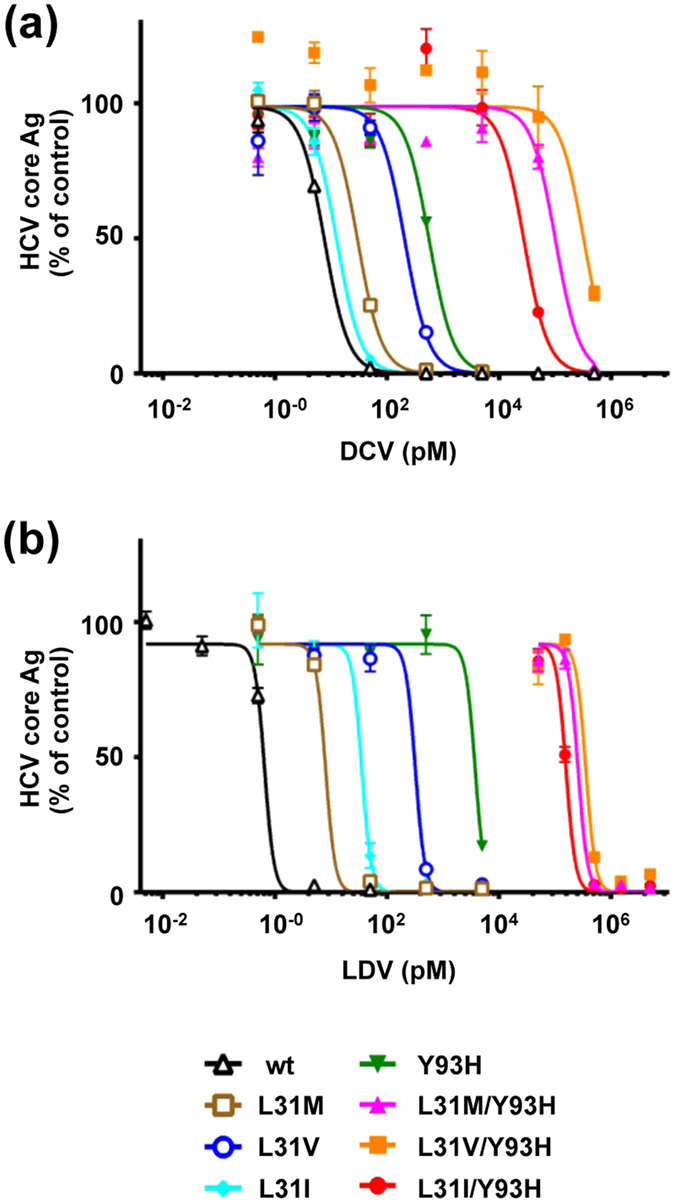
Susceptibility of JFH1/5ACon1 and its derivatives with resistance-associated NS5A mutations to NS5A inhibitors. Huh-7.5.1 cells were electroporated with *in vitro* transcribed HCV RNA. Four hours after electroporation, the culture media were replaced with fresh media containing DCV (**a**) or LDV (**b**). After incubation for 72 hours, the cells were harvested and intracellular HCV core Ags were measured. The data are presented as percentages of the DMSO-treated control.

We assessed the susceptibilities of NS5A recombinant viruses with resistance-associated NS5A mutations to the NS5A inhibitors DCV and Ledipasvir (LDV) to verify the resistance levels of these mutations in subgenomic replicons in previous studies. HCV RNA-transfected cells were treated with DCV or LDV for 72 hours, and intracellular HCV core Ag levels were measured and used to determine the effective concentrations required to inhibit 50% (EC50) values. HCV core Ag production of all recombinant viruses was inhibited at various levels following treatment with DCV or LDV as shown in Fig. 2. The EC50 value of JFH1/5ACon1-wt for DCV was 7.65 pM. Compared with JFH1/5ACon1-wt, variants with mutations at aa 31 (JFH1/5ACon1-L31M, -L31V, and -L31I) exhibited relatively low resistance (3.90-, 27.1-, and 1.65- fold, respectively) (Table 1). Variants with the Y93H single mutation (JFH1/5ACon1-Y93H) exhibited severe resistance (74.0-fold). When this mutation was combined with the mutation at aa 31 (JFH1/5ACon1-L31M/Y93H, -L31V/Y93H, and -L31I/Y93H), the resistance levels to DCV were strongly enhanced (13300-, 42700-, and 3640-fold, respectively), which was consistent with previous reports^9^,^10^. The resistance levels of the variants to LDV showed a tendency similar to the results obtained with DCV (Fig. 2b). However, the EC50 value of JFH1/5ACon1-wt for LDV (0.651 pM) was approximately 10-fold lower than DCV (7.65 pM), whereas the EC50 values of variants with Y93H to LDV were higher than those obtained with DCV. The EC50 values of JFH1/5ACon1-Y93H and -L31I/Y93H to LDV (3740 pM and 156000 pM, respectively) were remarkably higher compared with those to DCV (566 pM and 27800 pM, respectively) (Table 1). These results confirmed that the reported RAVs with the NS5A mutation conferred resistance to NS5A inhibitors and that these recombinant viruses could be useful for the evaluation of susceptibility to anti-HCV reagents.

### Exploration of effective anti-HCV reagents for variants with resistance- associated NS5A mutations

**Figure 3:**
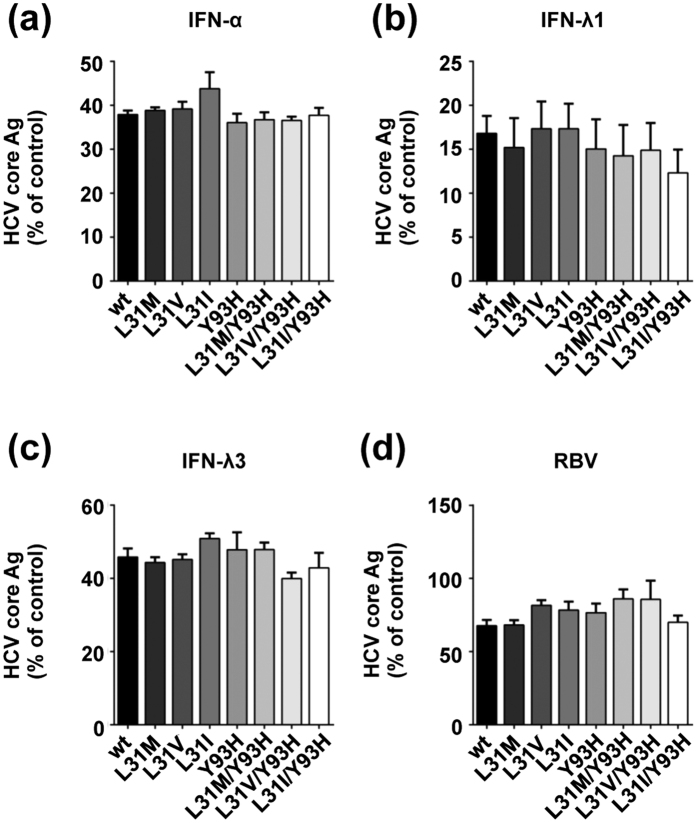
Susceptibility of JFH1/5ACon1 and its derivatives with resistance-associated NS5A mutations to IFNs and RBV. Huh-7.5.1 (**a–c**) or ORL8c (**d**) cells were electroporated with *in vitro* transcribed HCV RNA. Four hours after electroporation, the culture media were replaced with fresh media containing the indicated anti-viral reagents. After incubation for 72 hours, the cells were harvested and the intracellular HCV core Ags were measured. HCV core Ag levels of cells treated with IFN-α (10 IU/mL), IFN-λ1 (5 ng/mL), IFN-λ3 (1 ng/mL) and RBV (30 μM) are presented as percentages of the water- or DMSO-treated control.

To explore effective anti-HCV reagents against variants with resistance-associated NS5A mutations, we assessed the susceptibilities of JFH1/5ACon1 and its derivatives to IFN-α, -λ1 -λ3 and RBV. Seventy-two hours after treatment with IFN-α, -λ1, -λ3 and RBV, the intracellular HCV core Ag levels were measured in HCV RNA-transfected cells and the EC50 values were determined. For the RBV treatment analysis, we used ORL8c cells because the anti-HCV activities of RBV were reported to be precisely detected in Li23-derived cell lines but not in HuH-7-derived cell lines^25^,^26^. The production of HCV core Ag in these transfected cells was inhibited by IFNs and RBV in a concentration-dependent manner (Supplementary Figure S1). The EC50 values of JFH1/5ACon1-wt to IFN-α, -λ1, -λ3 and RBV were 4.64 IU/mL, 3.61 ng/mL, 0.711 ng/mL, and 24.1 μM, respectively (Table 2). The inhibition levels of intracellular HCV core Ag treated with these reagents were comparable at concentrations that were approximate to their EC50 values (Fig. 3). Therefore, the susceptibilities of the variants with resistance-associated NS5A mutations to IFNs and RBV were similar to those of JFH1/5ACon1-wt.

**Figure 4:**
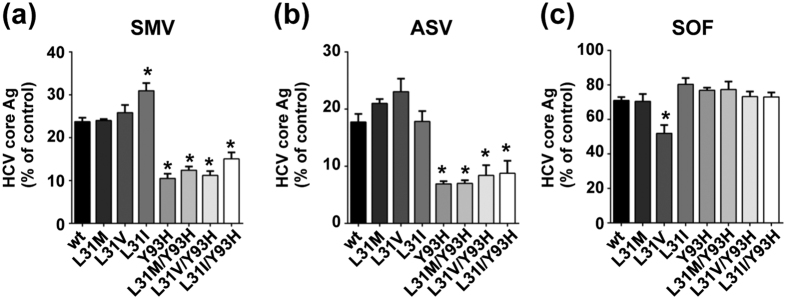
Susceptibility of JFH1/5ACon1 and its derivatives with resistance-associated NS5A mutations to protease inhibitors and a polymerase inhibitor. Huh-7.5.1 cells were electroporated with *in vitro* transcribed HCV RNAs. Four hours after electroporation, the culture media were replaced with fresh media containing the indicated anti-viral reagents. After incubation for 72 hours, the cells were harvested and the intracellular HCV core Ag was measured. HCV core Ag levels of cells treated with SMV (300 nM), ASV (1000 nM), and SOF (300 nM) are presented as percentages of the DMSO-treated control. Statistical significances to JFH1/5ACon1-wt are indicated (*p < 0.05).

Next, we examined the effects of protease inhibitors and a polymerase inhibitor on variants with resistance-associated NS5A mutations. Following treatment with Simeprevir (SMV), Asunaprevir (ASV), or Sofosbuvir (SOF), the production of HCV core Ag in these transfected cells was inhibited in a concentration-dependent manner (Supplementary Figure S1). HCV core Ag production by these strains was suppressed to various levels following treatment with the protease inhibitors ASV and SMV (Table 3). All strains exhibited higher susceptibilities to SMV compared with ASV. Interestingly, the HCV core Ag levels of variants with Y93H (5ACon1-Y93H, -L31M/Y93H, -L31V/Y93H, and -L31I/Y93H) were approximately 2-fold lower than those of JFH1/5ACon1-wt following treatment with 300 nM SMV and with 1000 nM ASV (Fig. 4a,b). The EC50 values of variants with Y93H (JFH1/5ACon1-Y93H, -L31M/Y93H, -L31V/Y93H, and -L31I/Y93H) to SMV and ASV were approximately one half (0.425- to 0.686-fold) of the EC50 values of JFH1/5ACon1-wt (Table 3). The susceptibilities of variants with a single mutation at aa 31 (JFH1/5ACon1-L31M, -L31V, and -L31I) to SMV and ASV were similar to JFH1/5ACon1-wt (Table 3). These findings suggested that second generation protease inhibitors such as SMV or ASV had strong anti-HCV potency against variants with Y93H. The EC50 value of JFH1/5ACon1-L31V (303 nM) to SOF was lower than that of the -wt (539 nM); the HCV core Ag levels of -L31V were also lower than those of -wt at the 300 nM concentration (Table 3, Fig. 4c). There were no significant differences in the EC50 values of the other strains to SOF. In our experiments, we found that variants with the Y93H and L31V mutations were more susceptible to the protease inhibitors and polymerase inhibitor, respectively, although remarkable differences were not observed in these strains following treatment with IFNs and RBV.

## Discussion

In the present study, we evaluated the characteristics of RAVs to NS5A inhibitors using the JFH1 based chimeric virus JFH1/5ACon1, which harbors NS5A from Con1 (genotype 1b). This system enabled the precise evaluation for effects of resistance-associated mutations on viral life cycle and for susceptibilities to anti-viral reagents. We found higher production of HCV core Ag in the culture medium of cells transfected with the chimeric virus with the Y93H mutation (JFH1/5ACon1-Y93H, -L31M/Y93H, -L31V/Y93H, and -L31I/Y93H) and lower production in the culture medium of cells transfected with chimeric viruses with L31V and L31I (Fig. 1b,c), suggesting that these mutations affected HCV production or propagation. Using the single-cycle virus production assay, we found that the intracellular HCV core Ag was increased in cells transfected with strains with the L31M mutation than in cells transfected with JFH1/5ACon1-wt, suggesting the enhancement of HCV replication by this mutation. HCV core Ag levels in JFH1/5ACon1-L31V and -Y93H-transfected cells were slightly lower and those in -L31V/Y93H-transfected cells were much lower than those in the -wt, indicating an additive effect of the L31V and Y93H mutations on the impairment of HCV replication (Fig. 1d). However, we also found that the intra- and extracellular infectivity titers were higher in variants with the Y93H mutation than in JFH1/5ACon1-wt, suggesting enhancement of infectious virus production by this mutation and a compensating effect of this mutation on the low replication efficiency. In comparison with the effect of Y93H on virus production, the effect of L31M was limited. In clinical studies, variants with the L31M and Y93H mutation are found in 2.7% to 3.3% and 8.5% to 23.2% of treatment naive patients, respectively^12^,^13^,^14^,^15^. Besides, unlike other variants resistant to IFN or protease inhibitors, variants with this mutation do not disappear and remain for a long time even after the cessation of NS5A inhibitor treatment in patients who fail treatment^9^,^17^,^18^. Moreover, the HCV RNA titers in patients infected with variants with the Y93H mutation have been reported to be higher than those in patients infected with variants without the Y93 mutation^27^,^28^,^29^,^30^. These observations indicate advantages of this mutation in HCV propagation. We hypothesized that this advantage could be explained by the mutation-associated enhancement of infectious virus production shown in Fig. 1e.

By exploiting this system, we explored the effectiveness of anti-HCV reagents against variants with resistance-associated NS5A mutations To evaluate the validity of the system for effective reagent screening, we examined the susceptibilities of these variants to NS5A inhibitors. In accordance with previous reports, JFH1/5ACon1-wt was susceptible to DCV and LDV compared with variants with mutations. Moreover, various resistance levels were observed following the introduction of mutations at aa 31 and aa 93 individually or in combination. Interestingly, the EC50 of JFH1/5ACon1-wt for LDV was lower than the EC50 to DCV and the effects of these mutations were more pronounced in resistance to LDV compared with DCV, which was consistent with previous reports^31^,^32^. The resistance levels of variants with Y93H to NS5A inhibitors were higher than those obtained in previous reports using the HCV subgenomic replicon assay but were comparable to the results obtained in analyses with the HCV cell culture system in a previous report^11^. The EC50 of variants with Y93H to NS5A inhibitors in the cell culture system might be affected by the enhanced infectious virus production level of Y93H shown in this study. Therefore, we reasoned that this system was suitable for the evaluation of the susceptibility of these variants to anti-HCV reagents.

For the assessment of anti-HCV reagents, we selected the type I and type III IFNs. However, we did not detect different effects on variants with resistance-associated mutations as a result of the administration of type I (IFN-α) and type III (IFN-λ1 and -λ3) IFNs. We also evaluated the effect of RBV using ORL8c cells. This cell line has been reported to be suitable for assessments of the anti-HCV efficacy of RBV in cell culture. However, this effect was not detected in the HCV cell culture system with HuH-7 derived cells due to the lower adenosine kinase expression level^26^,^33^. Despite the use of this suitable system, we could not find any difference in the anti-HCV effects between JFH1/5ACon1-wt and the strains with mutations. Our results were consistent with the clinical observations of the virological response rates to peg-IFN plus RBV therapy, which were not affected by the presence of a strain with Y93H^34^.

Next, we assessed the anti-HCV effects of DAAs targeting other HCV regions and found that the protease inhibitors SMV and ASV suppressed the propagation of chimeric viruses with the Y93H mutation more efficiently compared with JFH1/5ACon1-wt and the strains with other mutations. Additionally, the polymerase inhibitor SOF potently inhibited the propagation of the strain with L31V. Differences in effects of protease and polymerase inhibitors among RAVs with NS5A mutations have not been reported in *in vitro* study to date. However, in accordance with our results, a deep sequencing analysis revealed that the reduction in HCV RNA was significantly greater for a strain with Y93H than for a strain without the Y93H mutation in patients undergoing triple SMV, peg-IFN and RBV combination therapy^27^. NS5A forms the replication machinery complex with NS3, NS4A, NS4B and NS5B, and the structure and function of this complex may be affected by resistance-associated mutations. Alterations in the replication machinery complex may be associated with the mutation-associated enhancement of HCV replication and virus particle production and the susceptibilities to other DAAs. Previous studies indicated that the cooperation and interaction of NS5A with NS3 was essential for virus assembly^35^,^36^, and NS5A was shown to bind and regulate the polymerase activity of NS5B^37^,^38^. These reports led us to speculate that the interactions between HCV non-structural proteins were associated with the different susceptibilities of variants with resistance-associated mutations to protease and polymerase inhibitors. Notably, the EC50s of protease and polymerase inhibitors and RBV in the present study were higher than those reported in previous studies^26^,^33^,^39^,^40^,^41^. Consistent with our results, treatment analysis using the HCV cell culture system with JFH1 showed higher EC50s to these inhibitors than those reported in the replicon assays, possibly due to differences in the systems^42^,^43^,^44^. The recent development of many DAAs provides us with various treatment regimens for HCV. However, the combination of anti-HCV reagents depends on non-clinical factors and thus the combinations of anti-HCV reagents used in the clinic are restricted. Based on our data, a regimen including a protease inhibitor might be one option for the treatment of RAVs with NS5A inhibitors.

In conclusion, we demonstrated that variants with the Y93H mutation in NS5A had some advantages for HCV propagation in the *in vitro* analysis of the HCV cell culture systems and that the efficiency of infectious virus production was enhanced by this mutation. This property of Y93H may result in relatively high expression in treatment naïve patients on DAA therapy as well as high HCV RNA levels and persist for a long duration in patients. However, based on our findings, anti-HCV reagent combination therapies including protease inhibitors can be expected to have greater efficacy for variants with the Y93H mutation. Thus, these treatment regimens will be options for the treatment of patients infected with RAVs.

## Materials and Methods

### Cell Culture

The HuH-7-derived cell lines Huh-7.5.1 (provided by Francis Chisari, Scripps Research Institute, La Jolla, CA, USA) and Huh7-25, which lacks CD81 expression, were cultured at 37 °C in a 5% CO_2_ environment in complete growth medium as described previously^45^. Li23-derived ORL8c cells were also maintained as described previously^25^,^46^.

### Plasmid Construction

The construction of the JFH-1 recombinant virus with the NS5A from the Con1 strain (JFH1/5ACon1) was described previously^21^. The resistance-associated NS5A mutations L31M, L31V, L31I, and Y93H were introduced into JFH1/5ACon1 individually or in combination by site-directed mutagenesis with appropriate primers.

### RNA Transfection and Quantification of HCV Core Antigen

*In vitro* transcribed full-length HCV RNAs of these plasmids were electroporated into Huh-7.5.1 or Huh7-25 cells. Seventy-two hours after transfection, the supernatants and cells were harvested and used to measure the HCV core Ag. The concentration of the HCV core Ag was measured by the Lumipulse Ortho HCV Ag kit (Ortho Clinical Diagnostics, Tokyo, Japan) according to the manufacturer’s instructions. The methods for *in vitro* RNA synthesis and electroporation were described previously^47^,^48^.

### Susceptibility Analysis for Antiviral Reagents

Huh-7.5.1 (3 × 10^6^) cells and ORL8c cells were electroporated with 3 μg of synthetic HCV RNA, suspended in 20 mL of complete growth medium, and seeded into 12-well plates. Four hours after electroporation, the culture media were replaced with fresh media containing various concentrations of anti-HCV reagents. Seventy-two hours after incubation, the cells were harvested and the HCV core Ags were quantified. The anti-HCV reagents used were as follows: IFN-α2b (Intron A, MSD K.K., Tokyo, Japan), IFN-λ1 (Peprotech, Rocky Hill, NJ, USA), IFN-λ3 (IL28B; R&D Systems, Inc., Minneapolis, MN, USA), RBV (Sigma-Aldrich, St. Louis, MO, USA), DCV (Axon Medchem BV, Groningen, Netherlands), SMV (Toronto Research Chemicals Inc., North York, ON, Canada), ASV (Chemscene LLC, South Brunswick Township, NJ, USA), LDV (GS5885; AdooQ Bioscience, Irvine, CA, USA), and SOF (PSI-7977; Chemscene LLC). The IFNs were dissolved in water, and the DAAs and RBV were dissolved in dimethyl sulfoxide (DMSO) and used at a 0.1% final concentration. Dose-response curves were fitted with the following model, and the EC50 values of intracellular core protein level were calculated using the GraphPad Prism 6 software (GraphPad Software, Inc, CA, USA): Y = Bottom + (Top-Bottom)/(1 + 10^((LogEC-X)*HillSlope)), where X is the concentration of the reagent, Y is the response expressed as the percentage of HCV core Ag related to the corresponding control, LogEC represents the LogEC50 control, and Top and Bottom represents the Y values at the top and bottom plateau of the fitted curve, respectively.

### Titration of HCV Infectivity

The HCV infectivity titers were measured by indirect immunostaining as described previously^45^. Briefly, Huh-7.5.1 cells seeded into 96-well plates were cultured overnight and infected with serially diluted inocula. The inocula were collected from the culture media or cells from HCV-RNA transfected Huh7-25 cells. Three days after infection, indirect immunostaining was performed with an anti-core antibody (clone C7-50; Abcam, Cambridge, UK). The infectivity titer was expressed as the focus-forming units (FFU) per mL.

### Statistical Analysis

Statistical analysis was performed by an unpaired two-tailed Student’s t-test with Welch’s correction. P values less than 0.05 were considered statistically significant.

## Additional Information

**How to cite this article**: Nitta, S. *et al.* Effects of Resistance-Associated NS5A Mutations in Hepatitis C Virus on Viral Production and Susceptibility to Antiviral Reagents. *Sci. Rep.* **6**, 34652; doi: 10.1038/srep34652 (2016).

## Supplementary Material

Supplementary Information

## Figures and Tables

**Table 1:** EC50 values of JFH1/5ACon1 and its derivatives with resistance-associated NS5A mutations to DCV and LDV.

Strain (JFH1/5ACon1-)	DCV		LDV	
	EC50 (pM) (95% CI)	Fold Resistance	EC50 (pM) (95% CI)	Fold Resistance
wt	7.65 (5.61–10.4)	1.00	0.651 (0.560–0.757)	1.00
L31M	29.8 (15.1–44.7)	3.90	8.01 (6.03–9.96)	12.3
L31V	207 (81.1–333)	27.1	320 (199–441)	491
L31I	12.6 (5.61–19.7)	1.65	35.4 (24.3–46.4)	54.4
Y93H	566 (347–789)	74.0	3740 (2690–4800)	5750
L31M/Y93H	102000 (52900–151000)	13300	258000 (189000–326000)	396000
L31V/Y93H	327000 (173000–480000)	42700	353000 (244000–462000)	542000
L31I/Y93H	27800 (13300–42200)	3640	156000 (133000–180000)	240000

DCV; Daclatasvir, LDV; Ledipasvir, EC50; effective concentrations required to inhibit 50% of intracellular core protein level, CI; confidence interval.

**Table 2:** EC50 values of JFH1/5ACon1 and its derivatives with resistance-associated NS5A mutations to IFN-α, IFN-λ1, IFN-λ3 and RBV.

Strain (JFH1/5ACon1-)	EC50 (95% CI)			
	IFN-α (IU/mL)	IFN-λ1 (ng/mL)	IFN-λ3 (ng/mL)	RBV (μM)
wt	4.64 (2.88–7.48)	3.61 (2.39–5.46)	0.711 (0.461–1.10)	24.1 (15.3–38.0)
L31M	5.66 (2.13–9.19)	2.36 (1.04–3.68)	0.739 (0.316–1.17)	22.5 (16.1–28.9)
L31V	6.96 (2.64–11.3)	1.74 (0.801–2.67)	0.718 (0.306–1.13)	29.4 (21.2–37.8)
L31I	7.89 (2.99–12.8)	1.97 (0.892–3.06)	1.02 (0.436–1.61)	26.0 (18.7–33.3)
Y93H	7.19 (2.72–11.7)	2.50 (1.08–3.90)	0.853 (0.364–1.34)	24.8 (17.8–31.6)
L31M/Y93H	7.38 (2.79–12.0)	3.53 (1.52–5.56)	1.07 (0.458–1.69)	33.3 (24.0–42.7)
L31V/Y93H	5.66 (2.14–9.19)	3.04 (1.30–4.80)	0.512 (0.217–0.803)	30.8 (22.2–39.5)
L31I/Y93H	7.61 (2.88–12.3)	2.27 (0.996–3.53)	0.882 (0.375–1.38)	23.9 (17.2–30.6)

IFN; interferon, RBV; Ribavirin, EC50; effective concentrations required to inhibit 50% of intracellular core protein level, CI; confidence interval.

**Table 3:** EC50 values of JFH1/5ACon1 and its derivatives with resistance-associated NS5A mutations to SMV, ASV and SOF.

Strain (JFH1/5ACon1-)	SMV		ASV		SOF	
	EC50 (nM) (95% CI)	Fold Resistance	EC50 (nM) (95% CI)	Fold Resistance	EC50 (nM) (95% CI)	Fold Resistance
wt	173 (142–210)	1.00	664 (541–814)	1.00	539 (465–624)	1.00
L31M	187 (137–237)	1.08	684 (503– 870)	1.03	503 (404–604)	0.934
L31V	158 (117–200)	0.915	717 (533–903)	1.08	303 (244–363)	0.563
L31I	197 (146–247)	1.14	633 (456–810)	0.953	582 (466–695)	1.08
Y93H	110 (87.0–133)	0.638	291 (221–361)	0.438	663 (530–792)	1.23
L31M/Y93H	101 (79.6–122)	0.582	282 (214–350)	0.425	614 (493–733)	1.14
L31V/Y93H	118 (92.4–143)	0.681	374 (275–473)	0.563	561 (450–674)	1.04
L31I/Y93H	119 (92.9–144)	0.686	375 (276–475)	0.565	647 (521–776)	1.20

SMV; Simeprevir, ASV; Asunaprevir, SOF; Sofosbuvir, EC50; effective concentrations required to inhibit 50% of intracellular core protein level, CI; confidence interval.
