# Supplementary material for: Effects of Resistance-Associated NS5A Mutations in Hepatitis C Virus on Viral Production and Susceptibility to Antiviral Reagents
Source: Sci Rep. 2016 Oct 5;6:34652. doi: 10.1038/srep34652 (PMC5050404; doi:10.1038/srep34652)
Supplement: Supplementary Information [file srep34652-s1.pdf]

Supporting Information

Effects of Resistance-Associated NS5A Mutations in Hepatitis C Virus on Viral Production and Susceptibility to Antiviral Reagents

Sayuri Nitta, Yasuhiro Asahina, Mami Matsuda, Norie Yamada,

Ryuichi Sugiyama, Takahiro Masaki, Ryosuke Suzuki, Nobuyuki Kato,

Mamoru Watanabe, Takaji Wakita, Takanobu Kato.

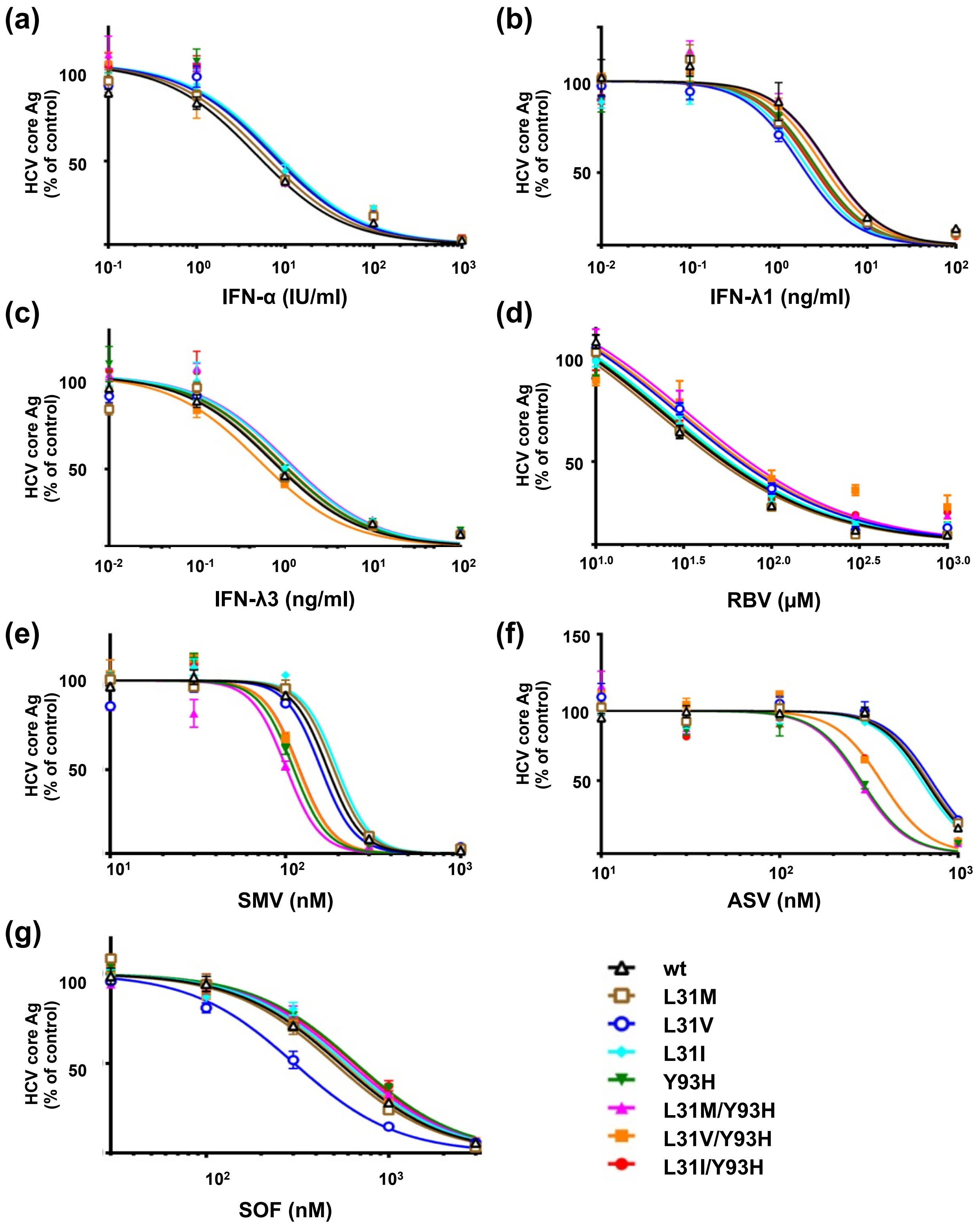
(a)
HCV core Ag (% of control)
100
50
10-1
10^0
10^1
10^2
10^3
IFN-α (IU/ml)
(b)
HCV core Ag (% of control)
100
50
10-2
10-1
10^0
10^1
10^2
IFN-λ1 (ng/ml)
(c)
HCV core Ag (% of control)
100
50
10-2
10-1
10^0
10^1
10^2
IFN-λ3 (ng/ml)
(d)
HCV core Ag (% of control)
100
50
10^1.0
10^1.5
10^2.0
10^2.5
10^3.0
RBV (μM)
(e)
HCV core Ag (% of control)
100
50
10^1
10^2
10^3
SMV (nM)
(f)
HCV core Ag (% of control)
150
100
50
10^1
10^2
10^3
ASV (nM)
(g)
HCV core Ag (% of control)
100
50
10^2
10^3
SOF (nM)
wt
L31M
L31V
L31I
Y93H
L31M/Y93H
L31V/Y93H
L31I/Y93H

Supplementary Figure S1. Susceptibility of JFH1/5ACon1 and its derivatives with resistance-associated NS5A mutations to IFNs, RBV and DAAs other than NS5A inhibitors.

Huh-7.5.1 (a -c, e - g) or ORL8c (d) cells were electroporated with *in vitro* transcribed HCV RNA. Four hours after electroporation, the culture media were replaced with fresh media containing IFN-α (a), IFN-λ1 (b), IFN-λ3 (c), RBV (d), SMV (e), ASV (f), and SOF (g). After incubation for 72 hours, the cells were harvested and the intracellular HCV core Ags were measured. The data are presented as percentages of the water- or DMSO-treated control.
